# A Study on the Evaluation of the Public Health Governance in Countries along the Belt and Road Initiative (BRI)

**DOI:** 10.3390/ijerph192214993

**Published:** 2022-11-14

**Authors:** Chenggang Zhang, Mingyu Wang

**Affiliations:** Department of Sociology, Tsinghua University, Beijing 100084, China

**Keywords:** social resilience, public health, risk governance, community of common health

## Abstract

Social resilience for public health is a new theoretical framework for understanding public health risk governance capacity. This study identifies 18 indicators from the publicly available database and adopts the method of multivariable analysis to evaluate the level of social resilience for public health in 73 countries along the Belt and Road Initiative (BRI). The study finds that the main influencing factors of social resilience for public health are institutional resilience, physical resilience, and participation resilience. The capacity of public health risk governance in countries along the BRI is classified into three levels: high, medium, and low. A high level of public health risk governance capacity is mainly shown in developed European countries such as Italy, rich Southwestern Asian countries such as the United Arab Emirates, and East Asian countries represented by Japan, South Korea, and Singapore. The middle-level countries are mainly distributed at the junction of Eurasia, which Russia represents. The low-level countries are primarily distributed in South Asia, Africa, and the war zones. In general, countries along the BRI have polarized the capacity of public health risk governance and structural inequalities, mainly manifested in large differences in the organizational capacity and mobilization capacity between countries in response to public health risk events, and the gap between the rich and the poor within a country. Under such circumstances, the building of a Community of Common Health is required to be accelerated.

## 1. Introduction

Health is considered the ultimate goal of reducing the risks of disasters [1]. Until 19 August 2022, the COVID-19 pandemic has caused a cumulative global caseload of 590.7 million cases and over 6.4 million deaths [2]. Humankind now lives in a pandemic risk society [3], with human health at an unprecedented risk [4].

Public health emergency is one of the most important elements of risk governance due to its uncertainty and hazards. Public health risk governance consists of many aspects, including public health risk identification [5,6], public health risk communication [7,8,9,10], and public health resilience construction [11,12,13,14,15,16,17]. “Resilience”, derived from the Latin word “resilio”, was specific to physical engineering to refer to the force that can restore an object to its original state after being subjected to an external force [18]. Afterwards, resilience research shifted from engineering to the socio-ecological domain [19,20].The definition of resilience evolved to include the ability to cope with or resist shocks, the adaptability that restores and reorganizes to establish the necessary function to minimalize and prevent catastrophic failure, and the transformative capacity to turn the uncertainty of a risky shock into an advantage [21].

In recent years, interest in building social resilience for health has been growing [22]. Many scholars have advocated that we should focus not only on the scientific character of the disease but also on the fundamental role of society when handling health risks [23]. The concept of social resilience was first used in 2000 by Adger, W.N., who introduced social influences to the original concept of resilience, and compared the differences between social and economic resilience at the community level. He defined social resilience as “the ability of communities to withstand external shocks to their social infrastructure” [24]. Following that, more scholars have analyzed the conceptual dimensions of social resilience, considering social resilience as the capability of society to cope, adapt, and transform as a whole in the face of disaster risks [25,26,27]. Social resilience for public health is the ability of health actors, institutions, and the public to work to prepare for and respond effectively to a crisis; the ability to maintain core functionalities when crisis strikes; and the ability to learn from the past crises and reorganize and transform when the condition requires [28]. Although the definition of social resilience for public health has been widely discussed, how to effectively build and measure health social resilience remains controversial.

The COVID-19 pandemic has hit both economic cooperation and health development for countries along the BRI. In the face of challenges posed by the pandemic, China has gradually developed a “health diplomacy” approach [29]. By developing “mask diplomacy” and the BRI healthcare infrastructure projects [30], China aims at improving the social resilience for public health when facing public health emergencies in countries along the BRI. Meanwhile, regional and global health studies increasingly demonstrate that economic prosperity cannot be achieved by relying on immense knowledge and a capacity gap exists in the public health system [31]. Understanding the level of social resilience for public health in the BRI countries is necessary due to their complicated national conditions, diversified environments, and public health governance capability. Through constructing a health vulnerability index for disaster risk reduction, Chan, E.Y.Y. [32] found three influencing factors of “population status”, “disease prevention”, and “coping capacity”, and figured out the vulnerability ranking of countries along the BRI from the perspective of disaster risk. However, vulnerability brings no constructive and responsive reflection. We should improve risk coping ability and understand the global capacity distribution under the guidance of social resilience for public health. Nevertheless, the existing public health risk governance evaluation lacks the social resilience for public health perspective. Moreover, the development indexes (such as HDI) created by the World Health Organization (WHO) and other international organizations do not comprehensively reflect the level of social resilience for public health in the BRI countries. A global unified system for evaluating the public health risk governance capacity of the Belt and Road region has not been established.

Compared with traditional research, social resilience for public health focuses on the complexity of public health, connecting health actors with non-health actors, providing a theoretical and practical framework for countries along the BRI to respond to public health emergencies, adaptation, and transformation, assessing the level of public health risk governance capacity of countries along the BRI.

## 2. Materials and Methods

Based on the theoretical framework of social resilience for public health, starting from the three dimensions of coping capacity, adaptable capacity, and transformative capacity, this paper constructs a set of social resilience for social health index to evaluate the level of social resilience for public health in countries along the BRI to characterize their public health risk governance capacity.

### 2.1. Construct the Social Resilience for Public Health Index

To evaluate the level of the public health risk governance capacity of countries along the BRI, this study selected 18 indicators from the three dimensions of coping ability, adaptability, and transformation ability based on the theoretical framework of social resilience and following the principle of index selection, which are (1) the reliability of indicator selection, referring to the reports of international organizations (e.g., WHO, UN) and the World Risk Index, and selecting public health-related indicators that can be reflected under the concept of social resilience; (2) the validity of indicator selection, ensuring the availability of data, which means open access to data from reliable sources (e.g., World Bank, WHO, etc.) to construct the Social Resilience for Public Health Index, as shown in Table 1.

### 2.2. Research Subjects and Data Sources

This study evaluates the level of public health governance capacity of the countries along the BRI. The data of the BRI countries, including Albania, France, Latvia, Japan, Ukraine, and China, were derived from the Social Sciences Academic Press (China) Database of Belt and Road Initiative [33].

The data of social resilience for public health indicators were obtained from publicly available databases, namely, World Development Indicators of the World Bank (WDI), Human Development Index (HDI) of the United Nations Development Programme (UNDP), Environmental Performance Index of the Yale Center for Environmental Law and Policy (EPI), and Fragile State Index of the Fund for Peace (FSI). According to the content of the indicators, data for the latest year with no missing data in the indicators are automatically selected, and missing data in the indicators are interpolated.

### 2.3. Statistical Analysis Method

To identify potential and unobserved common factors in health risk governance and assign weights to different indicators, we used the multivariate analysis method (Factor Analysis, FA) to Social Resilience for Public Health Index for dimension reduction. Based on the identification results of FA, the ranking level of public health risk management capabilities of countries along the BRI was obtained.

First, the data in the Social Resilience for Public Health Index go through positive transformation and standardization, and the applicability of the factor analysis methods was tested by Bartlett’s Sphericity Test and KMO Test. Bartlett’s Sphericity Test was used to test whether the relevant data array was a unit array, that is, whether the variables were independent. *p* = 0.00 in Bartlett’s test of sphericity in the results, which means that the original hypotheses were rejected, and the data matrix is not a unit array, there is a correlation between the variables and a common factor. The calculated KMO determines the correlation between variables by comparing the simple correlation coefficient and partial correlation coefficient among the variables. When the correlation is strong, the partial correlation coefficient is much smaller than the simple correlation coefficient, that is, the KMO value is close to 1. The calculated KMO of the data matrix is 0.87, indicating that it is suitable for factor analysis. To better explain the correlations between the factors, we used orthogonal rotation to obtain the results.

Next, the study measured the correlations between social resilience for public health factors and the Social Resilience for Public Health Index based on the number of loadings of each potential factor on the social resilience for public health indicators. The following are the four steps we followed in the study. (1) Identify the common factors. According to the factor eigenvalue in “Initial Eigenvalue” greater than 1, the common factors are extracted. (2) Calculate the weights of the three factors of different countries. According to the eigenvalues of the extracted factors, the weight scores of the factors of different countries are calculated automatically. (3) Calculate the country’s social resilience for public health score. Multiply the “Cumulative %”in “Rotation Square Sum” of the factors by the weight scores of the country, and then accumulate them to get the scores of the country. Higher values indicate stronger social resilience for public health of a country. (4) Classify the level of public health risk governance capacity of BRI countries into different levels based on the scores.

This study used IBM SPSS Statistics 27 (IBM, New York, NY, USA) for data cleaning and statistical analysis.

## 3. Results

### 3.1. The Social Resilience for Public Health Consists of Three Factor

This paper extracts common factors from Social Resilience for Public Health Index. Table 2 shows the contribution and the cumulative contribution of variance after orthogonal rotation of the factor analysis, representing the eigenvalues of each factor and the proportion of each factor variance in the total variance. The eigenvalues of Factor 1 are 4.855, accounting for more than 27% of the total variance, while the eigenvalues of Factor 2 and Factor 3 are 4.353 and 3.508 respectively, accounting for more than 15% of the total variance. The cumulative contribution of the variance of the first three factors is more than 70%, which basically covers the main information of the 18 indicators in the Social Resilience for Public Health Index. Next, the paper uses orthogonal rotation factors to obtain the rotation component matrix (as shown in Table 3). According to the cumulative contribution of variance in Table 2 and the matrix scores in Table 3, social resilience for public health consists of three structural influencing factors: institutional resilience, physical resilience, and participation resilience.

In this paper, we name Factor 1 as “institutional resilience.” In this factor, the coefficients of “T_R&D”, “C_LPI”, “C_PUBLIC”, “A_FSI”, “A_LIFE”, “C_PRIVATE” and “T_GII”, indicating that Factor 1 has commonality in the public service capacity of health care, which reflects a country’s medical supplies rescue efficiency, medical technology capabilities, public health development, the level of integrated vulnerability of healthcare, medical treatment level, medical organization collaboration, and national poverty gap. Institutional resilience is at the heart of health development and will be the focus of future global public health construction. In the face of a public health crisis, a country’s social resilience is largely determined by the effectiveness of institutional resilience, which is a comprehensive consideration of the economic, political, and social aspects of health care. The efficiency of medical transportation and rescue affects the emergency response capacity of medical care, search and rescue, and post-crisis reconstruction in health emergencies, and is the “first road” to achieve health social resilience. Medical technology is a key element to improve the social resilience for public health and an important foundation to promote the reform of the national health care system. A series of new medical ideas and concepts such as precision medicine, personalized medicine, and smart healthcare lay the foundation for institutional resilience. Social resilience for public health represents a comprehensive consideration of a social system, which is closely linked to the ecosystem in which it exists and incorporates aspects such as a country’s overall vulnerability and gender equality in reproductive health. Therefore, Factor 1 is common in national capacity, which reflects the integrity and organization of public health risk governance capacity, so it is named “institutional resilience”.

Factor 2 is named “physical resilience.” In Factor 2, indicators such as “A_SUPPORT”, “C_BASIC”, “C_WATER”, “A_POVERTY”, “C_PHYSICAL”, and “C_INTERNET” are strongly clustered, reflecting the conditions of a country’s citizens to face risks and indicating more about direct response capacity in terms of health risks. For example, a person’s degree of nutrition reflects his physical fitness in the face of risk; the availability of water and hospital beds in an area maintains the basic functioning of medical care for individuals; poverty and social support pressures represent the economic bottom line when dealing with risk; the Internet embodies the mutual assistance and interconnection of people. This factor directly affects the practical effect of public health risk management and is closely related to the individual body. Therefore, it is named as physical resilience.

Factor 3 is named “participation resilience.” The indicators of Factor 3, including “C_BED”, “T_GAP”, “C_PHYSICIAN”, “T_EI” and “A_EPI” are relatively more prominent and represents the degree of population vulnerability of a country, reflecting the level of the demographic structure of a country’s health social resilience, the structural social divide in health care, and the likelihood of social injustice. Within this factor, the structural characteristics of the population affect the level of participation in health care. This factor puts more emphasis on the internal structure of risk governance. For instance, if there are more beds and doctors in an area, more resources will be invested in risk governance. The higher the education level of a region, the higher the knowledge level of citizens’ participation in risk management, and the better the effect. Therefore, this factor is designated as participation resilience.

Meanwhile, the research calculates the scores of public health risk governance in countries along the BRI in terms of institutional, physical, and participation resilience characteristics and classifies the level of public health risk governance in countries along the BRI into three levels. Overall, countries along the BRI generally have low scores in institutional resilience, higher scores in physical resilience, and much room for improvement in participation resilience.

### 3.2. The Institutional Resilience of Countries along the BRI

The institutional resilience of public risk governance capacity in countries along the BRI is still weak. Systemic systems for coping with public health risk emergencies, such as transportation, medical science, and technology, health care system reform, and health care spending, have not yet been established. There are still gaps in various aspects of integrated vulnerability and gender equity in reproductive health in countries along the Belt and Road. For example, most of the countries along the BRI are developing countries, and there is less attention to gender issues in the field of reproductive health, meanwhile, basic gender health services such as gender education and gender medical checkups are weak.

In terms of the institutional resilience of public health risk governance capacity, the government’s public health expenditure is the major fiscal policy tool for intervening in health care development. According to “domestic general government health expenditure (% of GDP)” from World Bank Open Data, the emphasis on public health services varies significantly among the countries along the BRI. In 2019, the world average for government spending on public health was 5.89%, which is greatly surpassed by the developed countries along the BRI. For example, Japan reached 9.01%. In contrast to the developed countries where the advanced health care system serves the public in basic health, the South Asian countries spend less than 1% of their GDP on health averagely, thus the health of residents faces serious challenges. This data profile shows that the institutional resilience composed of public health expenditure has formed a fracture in developed countries and less developed countries, and the development of the current situation produces polarization. A severe problem of the COVID-19 pandemic is that public hospitals are severely “overcrowded” with COVID-19 patients, causing prominent structural conflict between the increase in the number of patients and the shortage of intensive care beds and doctors and nurses. Meanwhile, private health care spending significantly affects a country’s social resilience for public health. Private medical institutions can be the leading force in coordinating the problem of medical resource allocation. The government’s purchase of private medical services and the donation of medical supplied by private medical institutions can alleviate the problem of strained medical resources.

### 3.3. The Physical Resilience of Countries along the BRI

The physical resilience of public health risk governance capacity in countries along the BRI is relatively high, indicating that the medical infrastructure of the countries has been initially established, that basic sanitation services, basic drinking water services, and the use of internet can meet the basic needs of people’s lives. The countries have the basic resilience in the face of public health emergencies.

However, a large proportion of people in countries along the BRI are still not covered by primary medical care and universal education, and the quantity and quality of spatial coverage of physical resilience of public health still need to be reinforced. According to World Bank open data, from 2000 to 2020, the population using at least basic health services rose from 55% to 78%, and the world has seen steady growth in healthcare infrastructure. However, more than 20% of the world’s population still does not have access to basic health services. These individuals themselves may no longer be able to tolerate minor ailments, let alone risks associated with public health events. The countries such as Kenya and Timor-Leste still suffer from hunger, poor health care, and awful teaching conditions. Countries such as Afghanistan and Yemen still face localized wars, and the physical resilience of public health risk governance capabilities is extremely fragile.

### 3.4. The Participation Resilience of Countries along the BRI

The participation resilience scores in countries along the BRI are relatively low. Participation resilience is a common concern for countries around the world. On the one hand, the population structure constrains social resilience for public health. The number of medical workers and hospital beds cannot meet the risk management ability of public health. The ecological environment along the BRI is complex and fragile. Frequent natural disasters and deteriorating air environment seriously threaten individual physical health.

On the other hand, people in poor areas lack health guarantees and have increasingly prominent health problems due to various reasons such as economic, educational, and medical infrastructure. For example, the country of Nigeria has been in extreme impoverishment for a long time. According to World Bank open data, the percentage of the population living in extreme poverty (living on less than USD 1.90 per day) in Nigeria was 39.1%, much higher than the world average of 8.7% (2018). About 40% of the Nigerian population does not have access to adequate health treatment because of poverty, which has deprived Nigerians of the participatory nature of building public health resilience. During the COVID-19 pandemic, Nigeria’s social security was extremely precarious, with a combination of the pandemic virus, social violence, power corruption, hunger, and poverty impacting the participation resilience for health, and the COVID-19 death rate is extremely high. It is a social fact that public health care in Nigeria is poor and inadequate, yet the social instability brought about by poverty is a further impediment to the public’s motivation to address health risks jointly, as they live in chronic poverty and are unable to secure their basic livelihoods.

### 3.5. The Level of Public Health Governance Capacity Shows Regional Differences and Polarization

The level of public health risk governance capacity of the countries along the BRI is derived based on the factor weighing, and the capacity is classified into levels. The level of public health risk governance capacity of countries along the BRI can be classified as “high”, “medium”, and “low”. Among the countries of the BRI, there are 22 countries with strong public health risk governance capacity, such as Japan; 19 countries with medium governance level, such as Russia; and 32 countries with weak public health risk management capacity, such as Afghanistan.

In general, the level of public health risk governance in countries along the BRI is regionalized and polarized: there are more countries at the high and low ends of the spectrum, and the gap between the high and low levels is large. Countries with high levels of risk governance capacity have a good foundation of institutional and physical resilience, characterized by high levels of national economic development, medical technology, and public–private medical balance, and distributed mainly in developed European countries such as Italy, rich Southwestern Asian countries such as the United Arab Emirates, and East Asian countries represented by Japan, South Korea, and Singapore. Mainly concentrated on the intersection of Asia and Europe and represented by Russia, countries with a medium level of risk governance capacity have basically consolidated the physical resilience of public health risk governance. However, there is still room for progress in terms of institutional resilience and participation resilience and the need to pay attention to the construction of public health care services and medical investment. Countries with low public health risk governance capacity lag in institutional resilience, physical resilience, and participation resilience. Azerbaijan, Turkmenistan, Syria, Armenia, and Georgia rank the worst in institutional resilience. Kenya, Timor-Leste, Afghanistan, Cambodia, and India still lag in physical resilience, and Nigeria, Afghanistan, Yemen, Timor-Leste, and Kenya have the lowest levels of resilience. Based on this, countries with low public health risk governance capacity are mainly located in war zones, South Asia, and Africa.

## 4. Discussion and Conclusions

Understanding the public health risk governance capabilities of countries along the BRI requires a shift from “responding to risks” to “building social resilience”. First, the public health risk governance capacity of countries along the BRI is influenced by institutional resilience, physical resilience, and participation resilience, among which institutional resilience plays the most significant role. The institutional resilience of countries along the BRI is unevenly developed. The BRI countries generally lag in institutional resilience except for the developed European countries. Countries along the BRI are basically capable of handling the risk of public health emergencies, but the physical resilience of less developed countries and countries in war zones needs to be strengthened in terms of quantity and quality. The participation resilience of countries along the BRI faces the common threats of trend of low-birth rate and structural inequality. Second, the level of public health risk management of countries along the BRI demonstrates regional differences and polarization, with relatively large number of countries at the high and low ends of the spectrum and differs largely in terms of risk governance capacity. Countries with high-risk governance capacity have good institutional and physical resilience, mainly shown in developed European countries such as Italy, rich Southwestern Asian countries such as the United Arab Emirates, and East Asian countries represented by Japan, South Korea, and Singapore. Countries with a medium level of risk governance capacity have more room for progress in institutional and participation resilience, mainly in the junction of Eurasia represented by Russia. Countries with low-risk governance capacity lag in institutional resilience, physical resilience, and participation resilience, mainly concentrated in the war zones, South Asia, and Africa. Third, far from being any single country’s problem, addressing public health risks requires a global effort to eliminate health inequities.

For a long time, social resilience has been considered an aspect of social vulnerability, leading to the persistent neglect of it [34]. Actually, social resilience is neither the opposite of social vulnerability nor its element [35]. When responding to risk, social resilience and social vulnerability are both exposed to pressures from socio-economic and socio-environmental systems [36]. However, social resilience shows an enduring, adaptable and transformative advantage in risk governance that can go beyond vulnerability to tap into people’s epistemological content [37]. This is because the conceptual dimension of social resilience carries with its functional attributes that enable effective responses to various types of risks [38,39,40,41]. Humankind can effectively resist the impact of disaster risk within a framework of social resilience.

Some scholars attach importance to the construction of social resilience for public health. Morton, M.J. and Lurie, N. [42] argued that constructing true social resilience requires a fundamental expansion from a traditional approach, which emphasizes physical capacity building and material disaster relief, to long-term social capacity building. However, traditional public health risk governance is spatially compartmentalized, and the social resilience system has not yet been established. In the case of the COVID-19 pandemic, the response capacity of residential living spaces has been relatively weak because most medical resources are devoted to public hospitals. The pandemic prevention and control measures such as spatial lockdowns, social isolation has caused economic downturns, and people at the bottom of society have suffered a huge economic impact. This group is increasingly less able to obtain stable food supplies and health security due to their lack of hygiene knowledge, difficulty in accessing information, poor living conditions and declining household incomes. In the meantime, some scholars have given the countermeasures of social resilience for public health in empirical research. Hanefeld et al. [43] examined the health system by Ebola Virus Disease (EVD), pointing out three functional dimensions of the resilience of health system, namely “health information systems” (having the information and the knowledge to decide on what needs to be carried out), “funding/financing mechanisms” (investing or mobilizing resources to fund a response); and “health workforce” (who should plan and implement it and how). Based on their analysis of the COVID-19 experiences, Sundararaman, et al. [44] argued that social resilience plays a role from five dimensions of primary health care, surge capacity, health management information systems, medical technology, and governance. While the world’s institutional resilience for health is still weak. Most countries have responded to the COVID-19 pandemic with public health care. Therefore, people at the social bottom have been forced to withhold publicly funded health care or pay more to access essential private health services when the public services are halted. In most cases, under such health inequities, the underprivileged have been excluded from the health care system due to delays in accessing health care and lack of continuing care, making access to health care much more difficult.

The social resilience for public health is largely related to social equality with structural characteristics. Equality in health is more of a normative concept, that is, health inequality is a socially derived process that greatly affects the composition of social resilience in public health. We should be aware that although the level of medical technology and epidemic prevention technology around the world has improved to varying degrees, the social gap in health has widened. Only by meeting the medical needs of the rich can the poor be able to obtain health care [45]. The COVID-19 pandemic precisely exposes this long-standing structural conflict in health inequalities [46]. For example, in India, a government-led program to provide universal health care coverage for the Indian population was introduced as early as 1946 and is known for its “medical tourism”. However, after half a century, due to various reasons such as the large population and poverty in India, there is still a large gap in the quality of public health care facilities in India, with health care resources unevenly distributed and of varying quality. India’s health care coverage is severely inadequate in remote rural areas, with a severe shortage of human resources for health and a shortage of qualified health workers. In 2019, India’s physician coefficient per 1000 people was only 0.9, in stark contrast to its advanced private healthcare. In 2021, India’s social security in the COVID-19 pandemic is threatened by the dual threat of viral mutation and social instability, which further indicates the fragility of social resilience for public health. The poor quality of health care facilities exemplified by India is a problem that we can see also in other Asian countries such as Cambodia and Laos, whose health societies are not resilient enough to withstand any public health risks.

In summary, the differences in public health risk governance capacity of countries along the BRI are manifested in health inequalities. From the perspective of breadth, there are structural conflicts in health inequality in developed, developing, and underdeveloped regions. The health care expenditure and medical infrastructure level of developed countries are much higher than those of developing countries and less developed countries. From the perspective of depth, structural factors such as differences in gender, population, education, and wealth within countries increase health inequalities. This requires a global effort to promote the construction of a global community of health for all. According to this study, eliminating health inequalities and building a global community of health for all is an irresistible trend of global health governance. The development path of building a global community of health for all is to strengthen the resilience of the medical and health care system. The foundation of its development is to enhance the physical resilience of medical infrastructure and services. The essence of its development is to guarantee the safety of human life and enhance the participation resilience of public health risk governance.

There are certain limitations to this study. First, in terms of research content, this study focuses on the use of health social resilience to characterize public health risk governance capacity, which is achieved by constructing a health social resilience index to measure. This measurement focuses on the social macro-level of national health care. However, a country’s health social resilience is not related to its ability to cope, adapt, and transform at the macro level, but is also dynamic at the meso and micro levels in terms of institutional systems, organizational structures, social capital, and family structures, but the latter is not focused on and measured. Second, from the methodological point of view, the number of sample countries and regions is limited, and several indicators have not been updated to the latest year. There are missing values in the data of sample countries, which are measured by interpolation, and thus there may be errors in the ranking of social resilience for public health, which shall only be used as a reference for national public risk governance capacity.

## Figures and Tables

**Table 1:** Key indicators and correlation of Social Resilience for Public Health Index.

Dimension	Indictors (Abbreviation)	Explanation of the Content of Social Resilience for Public Health	Correlation	Source	Number
Coping capacity (C)	Availability of Physicians (C_PHYSICIAN)	“C_PHYSICIAN” measures the social resilience for public health by “physicians (per 1000 people)” to evaluate the social resilience for public health. This indicator shows the number of participants in primary health care meeting the social resilience for health care by calculating data on health worker (physicians, nurses and midwives, and community health workers) density. This reflects a region’s public health risk governance participation ability.	+	WDI	1
	Hospital Beds (C_BED)	“C_BED” measures hospital beds (per 1000 people), which basically reflects the bed capacity of a country’s national health system. The indicator includes inpatient beds available in public, private, general, specialized hospitals, and rehabilitation centers. This also reflects a region’s public health risk governance participation ability.	+	WDI	2
	Basic Sanitation Services (C_BASIC)	“C_BASIC” takes “people using at least basic sanitation services (% of the population)” to evaluate the social resilience for public health and observe individual access to basic health services in a country.	+	WDI	3
	Public Medical Attention. (C_PUBLIC)	“C_PUBLIC” measures “domestic general government health expenditure (% of GDP)”, which shows the overall importance a country attaches to public health.	+	WDI	4
	Development Level of Private Health (C_PRIVATE)	“C_PRIVATE” measures “domestic private health expenditure (% of current health expenditure)”, showing the possibility that government and private sector cooperates in health, which represents the mobilization ability to deal with public health risk events.	+	WDI	5
	Logistics Performance of Health (C_LPI)	“C_LPI” adopts the “Logistics Performance Index”, which shows a country’s reflexes and organizing abilities in response to public health emergencies.	+	WDI	6
	Health Networking Level (C_INTERNET)	“C_INTERNET” considers “individuals using the Internet (% of the population)”. It reflects the individual’s ability to access information and social solidarity in response to public health risk events.	+	WDI	7
	Satisfaction of Medical Water (C_WATER)	“C_WATER” evaluates “people using at least basic drinking water services (% of the population).” It reflects individual access to water resources to sustain life.	+	WDI	8
	Physical Resistance (C_PHYSICAL)	“C_PHYSICAL” uses the “prevalence of undernourishment (% of the population)”. Inadequate food intake fails to meet basic dietary energy needs, making individuals more challenging when coping with complex health risks.	-	WDI	9
Adaptive capacity (A)	Degree of Fragile State (A_FSI)	“A_FSI” adopts the “Fragile State Index”, which shows a country’s comprehensive adaptability in economic, political, and social terms when facing health risks.	-	FSI	10
	Health Poverty (A_POVERTY)	“A_POVERTY” adopts “poverty headcount ratio at national poverty lines (% of the population)”, reflecting people’s economic adaptability in health risk. The higher the degree of poverty, the more difficult it is for them to adapt to health risks.	-	WDI	11
	Social Support Pressure (A_SUPPORT)	“A_SUPPORT” takes “age dependency ratio (% of working-age population)” to show a country’s demographic adaptation pressures in the face of health risks. The higher the degree of support, the more difficult it is for them to adapt to health risks.	-	WDI	12
	Life Expectancy (A_LIFE)	“A_LIFE” adopts “life expectancy at birth, total (years)” to show a country’s comprehensive medical level.	+	WDI	13
	Environmental Performance (A_EPI)	“A_EPI” adopts the “Environmental Performance Index” jointly published by Yale and Columbia, reflecting a country’s environmental health and ecosystem vitality. The environment affects the health of individuals, and indirectly affects residents’ participation in dealing with public health risk events.	+	EPI	14
Transformative capacity (T)	Medical Research and Development Capabilities (T_R&D)	“T_R&D” measures “research and development expenditure (% of GDP)”, which observes the basic research, applied research, and experimental development capabilities of enterprises, government, higher education sector, and private non-profit organizations.	+	WDI	15
	Social Gap between Rich and Poor (T_GAP)	“T_GAP” adopts the “Gini Index”, to reflect the income distribution gap of a country, as well as the differences and fairness of people participating in public health risk governance.	-	WDI	16
	Social Education Level (T_EI)	“T_EI” uses the “mean years of schooling” of “Education Index” to show the social education level in social resilience and judge the level of public health knowledge that the public can understand.	+	HDI	17
	Gender Reproductive Health (T_GII)	“T_GII” uses the “Gender Inequality Index (GII)” to evaluate three aspects of a country: reproductive health, empowerment, and economic status. It reflects the gender gap in health in society. Thus, the higher the GII value, the more disparities between females and males and the more loss to human development, which is detrimental to the sustainable development of social resilience for public health.	-	HDI	18

**Table 2:** Results of factor analysis.

Factor	Initial Eigenvalue			Rotation Square Sum		
	Eigenvalue	Variance%	Cumulative %	Eigenvalue	Variance%	Cumulative %
Factor 1	9.222	51.231	51.231	4.855	26.975	26.975
Factor 2	2.157	11.984	63.214	4.353	24.181	51.156
Factor 3	1.338	7.433	70.648	3.508	19.491	70.648
Factor 4	0.953	5.293	75.940			
Factor 5	0.765	4.251	80.191			
Factor 6	0.635	3.528	83.719			
Factor 7	0.578	3.212	86.930			
Factor 8	0.422	2.345	89.275			
Factor 9	0.385	2.139	91.415			
Factor 10	0.347	1.929	93.343			
Factor 11	0.268	1.490	94.833			
Factor 12	0.214	1.187	96.019			
Factor 13	0.183	1.019	97.038			
Factor 14	0.144	0.801	97.839			
Factor 15	0.126	0.700	98.539			
Factor 16	0.117	0.649	99.188			
Factor 17	0.077	0.425	99.613			
Factor 18	0.070	0.387	100.000			

**Table 3:** The results of common factor extraction.

Variable	Factor 1	Factor 2	Factor 3
C_PHYSICIAN			0.681
C_BED			0.710
C_BASIC		0.780	
C_PUBLIC	0.762		
C_PRIVATE	−0.623		
C_LPI	0.793		
C_INTERNET		0.649	
C_WATER		0.757	
C_PHYSICAL		−0.682	
A_FSI	−0.733		
A_POVERTY		−0.749	
A_SUPPORT		−0.811	
A_LIFE	0.713	0.538	
A_EPI	0.576		0.635
T_R&D	0.820		
T_GAP			−0.697
T_EI			0.674
T_GII	−0.565		−0.559

Blanks represent abs (loading) < 0.5.

## Data Availability

(1) The data used in this study are collected in https://cloud.tsinghua.edu.cn/d/55ca584986e24c67912f/ (accessed on 4 November 2022); (2) Country data of the BRI came from Social Sciences Academic Press (China) Database of Belt and Road Initiative Country Available online: https://www.ydylcn.com/skwx_ydyl/sublibrary?ID=8728&SiteID=1&showDetail=true&RootFlag=Y (accessed on 6 February 2022).

## References

[1] Lo S.T.T., Chan E.Y.Y., Chan G.K.W., Murray V., Abrahams J., Ardalan A., Kayano R., Yau J.C.W. (2017). Health Emergency and Disaster Risk Management (Health-EDRM): Developing the Research Field within the Sendai Framework Paradigm. Int. J. Disaster Risk Sci..

[2] COVID-19 Response Fund WHO Coronavirus (COVID-19) Dashboard. https://covid19.who.int.

[3] Mansouri F., Sefidgarbaei F. (2021). Risk Society and COVID-19. Can. J. Public Health.

[4] Bhattacharjee A., Saha M., Halder A., Debnath A., Mukherjee O. (2021). Therapeutics and Vaccines: Strengthening Our Fight Against the Global Pandemic COVID-19. Curr. Microbiol..

[5] Perdue M.L., Swayne D.E. (2005). Public Health Risk from Avian Influenza Viruses. Avian Dis..

[6] Lupton D. (1993). Risk as Moral Danger: The Social and Political Functions of Risk Discourse in Public Health. Int. J. Health Serv..

[7] Bennett P., Calman K. (2001). Risk Communication and Public Health. Public Understand. Sci..

[8] Covello V.T. (2003). Best Practices in Public Health Risk and Crisis Communication. J. Health Commun..

[9] Glik D.C. (2007). Risk Communication for Public Health Emergencies. Annu. Rev. Public Health.

[10] WHO (2017). Communicating Risk in Public Health Emergencies: A WHO Guideline for Emergency Risk Communication (ERC) Policy and Practice.

[11] Khan Y., O’Sullivan T., Brown A., Tracey S., Gibson J., Généreux M., Henry B., Schwartz B. (2018). Public Health Emergency Preparedness: A Framework to Promote Resilience. BMC Public Health.

[12] Panter-Brick C. (2014). Health, Risk, and Resilience: Interdisciplinary Concepts and Applications. Annu. Rev. Anthropol..

[13] Seaman P., McNeice V., Yates G., McLean J. (2014). Resilience for Public Health. Glasg. Cent. Popul. Health.

[14] Ziglio E., Azzopardi-Muscat N., Briguglio L. (2017). Resilience and 21st Century Public Health. Eur. J. Public Health.

[15] Zautra A.J., Hall J.S., Murray K.E. (2010). Resilience: A New Definition of Health for People and Communities. Handbook of Adult Resilience.

[16] Wulff K., Donato D., Lurie N. (2015). What Is Health Resilience and How Can We Build It?. Annu. Rev. Public Health.

[17] Wister A., Speechley M. (2020). COVID-19: Pandemic Risk, Resilience and Possibilities for Aging Research. Can. J. Aging/La Rev. Can. Du Vieil..

[18] Klein R.J.T., Nicholls R.J., Thomalla F. (2003). Resilience to Natural Hazards: How Useful Is This Concept?. Environ. Hazards.

[19] Holling C.S. (1973). Resilience and Stability of Ecological Systems. Annu. Rev. Ecol. Syst..

[20] Walker B., Holling C.S., Carpenter S.R., Kinzig A. (2004). Resilience, Adaptability and Transformability in Social–Ecological Systems. Ecol. Soc..

[21] Brown A., Dayal A., Rumbaitis Del Rio C. (2012). From Practice to Theory: Emerging Lessons from Asia for Building Urban Climate Change Resilience. Environ. Urban..

[22] Douglas M., Wildavsky A. (2010). Risk and Culture an Essay on the Selection of Technological and Environmental Dangers.

[23] Dagdeviren H., Capucha L., Calado A., Donoghue M., Estêvão P. (2020). Structural Foundations of Social Resilience. Soc. Policy Soc..

[24] Adger W.N. (2000). Social and Ecological Resilience: Are They Related?. Prog. Hum. Geogr..

[25] Lorenz D.F. (2013). The Diversity of Resilience: Contributions from a Social Science Perspective. Nat. Hazards.

[26] Shaw D., Scully J., Hart T. (2014). The Paradox of Social Resilience: How Cognitive Strategies and Coping Mechanisms Attenuate and Accentuate Resilience. Glob. Environ. Change.

[27] Paton D., Johnston D. (2017). Disaster Resilience: An Integrated Approach.

[28] Kruk M.E., Myers M., Varpilah S.T., Dahn B.T. (2015). What Is a Resilient Health System? Lessons from Ebola. Lancet.

[29] Buckley P.J. (2020). China’s Belt and Road Initiative and the COVID-19 Crisis. J. Int. Bus. Policy.

[30] Mouritz F. (2020). Implications of the COVID-19 Pandemic on China’s Belt and Road Initiative. Connections.

[31] Tambo E., Khayeka-Wandabwa C., Muchiri G.W., Liu Y.-N., Tang S., Zhou X.-N. (2019). China’s Belt and Road Initiative: Incorporating Public Health Measures toward Global Economic Growth and Shared Prosperity. Glob. Health J..

[32] Chan E.Y.Y., Huang Z., Lam H.C.Y., Wong C.K.P., Zou Q. (2019). Health Vulnerability Index for Disaster Risk Reduction: Application in Belt and Road Initiative (BRI) Region. Int. J. Environ. Res. Public Health.

[33] Social Sciences Academic Press (China) Database of Belt and Road Initiative_Country. https://www.ydylcn.com/skwx_ydyl/sublibrary?ID=8728&SiteID=1&showDetail=true&RootFlag=Y.

[34] Wisner B., Blaikie P., Cannon T., Davis I. (2014). At Risk: Natural Hazards, People’s Vulnerability and Disasters.

[35] Manyena S.B. (2006). The Concept of Resilience Revisited: The Concept of Resilience Revisited. Disasters.

[36] Adger W.N. (2006). Vulnerability. Glob. Environ. Change.

[37] Bankoff G. (2003). Cultures of Disaster: Society and Natural Hazards in the Philippines.

[38] Brown K. (2014). Global Environmental Change I: A Social Turn for Resilience?. Prog. Hum. Geogr..

[39] Revilla J.C., Martín P., Castro C. (2018). de The Reconstruction of Resilience as a Social and Collective Phenomenon: Poverty and Coping Capacity during the Economic Crisis. Eur. Soc..

[40] Hutter G. (2013). Organizing Social Resilience in the Context of Natural Hazards: A Research Note. Nat. Hazards.

[41] Maclean K., Cuthill M., Ross H. (2014). Six Attributes of Social Resilience. J. Environ. Plan. Manag..

[42] Morton M.J., Lurie N. (2013). Community Resilience and Public Health Practice. Am. J. Public Health.

[43] Hanefeld J., Mayhew S., Legido-Quigley H., Martineau F., Karanikolos M., Blanchet K., Liverani M., Yei Mokuwa E., McKay G., Balabanova D. (2018). Towards an Understanding of Resilience: Responding to Health Systems Shocks. Health Policy Plan..

[44] Sundararaman T., Muraleedharan V.R., Ranjan A. (2021). Pandemic Resilience and Health Systems Preparedness: Lessons from COVID-19 for the Twenty-First Century. J. Soc. Econ. Dev..

[45] Victora C.G., Vaughan J.P., Barros F.C., Silva A.C., Tomasi E. (2000). Explaining Trends in Inequities: Evidence from Brazilian Child Health Studies. Lancet.

[46] Ahmed F., Ahmed N., Pissarides C., Stiglitz J. (2020). Why Inequality Could Spread COVID-19. Lancet Public Health.

